# DAPT, a γ-Secretase Inhibitor, Suppresses Tumorigenesis, and Progression of Growth Hormone-Producing Adenomas by Targeting Notch Signaling

**DOI:** 10.3389/fonc.2019.00809

**Published:** 2019-08-27

**Authors:** Jie Feng, Jianpeng Wang, Qian Liu, Jiye Li, Qi Zhang, Zhengping Zhuang, Xiaohui Yao, Chunhui Liu, Yangfang Li, Lei Cao, Chuzhong Li, Lei Gong, Dan Li, Yazhuo Zhang, Hua Gao

**Affiliations:** ^1^Key Laboratory of Central Nervous System Injury Research, Center of Brain Tumor of Beijing Institute for Brain Disorders, Beijing Neurosurgical Institute, Capital Medical University, Beijing, China; ^2^The Affiliated Hospital of Medical College, Qingdao University, Qingdao, China; ^3^Surgical Neurology Branch, National Institute of Neurological Disorders and Stroke, National Institutes of Health, Bethesda, MD, United States; ^4^Neurosurgery, Shanxi Provincial People's Hospital, Taiyuan, China

**Keywords:** pituitary adenoma, growth hormone-producing adenomas, Notch signaling, inhibitor, DAPT, invasion

## Abstract

Advances in the understanding of growth hormone-producing adenomas (GHomas) are ongoing, but current therapy is limited by moderate and variable efficacy and in need of life-long treatment. In this study, the molecular signaling pathway related to GHoma was investigated by proteomics and transcriptomics. The differentially expressed proteins and genes were significantly enriched in Extracellular Matrix-Receptor Interactions, Notch Signaling, Basal Cell Carcinoma Signaling, JAK-STAT3, Wnt Signaling, and Glioblastoma Multiforme Signaling by Ingenuity Pathway Analysis. Furthermore, the Notch2/Delta-like canonical Notch ligand (DLL) signaling pathway was identified to be associated with tumorigenesis and invasiveness of GHoma. In 76 patients, Notch2 and DLL3 were upregulated in invasive compared to those in non-invasive GHoma (*p* < 0.05). Disease-free survival was significantly longer in patients with low, compared with high, DLL3 expression (*p* = 0.027). Notch 2 knockdown inhibited cell migration in both GH3 cells and primary GHoma cells, along with downregulation of the mRNA expression of related genes. DAPT, a γ-secretase inhibitor, inhibited tumor growth and invasion *in vivo* and *in vitro* and suppressed the release of growth hormone in primary GHoma cells. The involvement of Notch2/DLL3 signaling in GHoma progression warrants additional study of Notch inhibitor, DAPT, as a potential GHoma treatment.

**Importance of the Study:**

Current treatments of GH adenomas (GHomas) are limited by their moderate and variable efficacy and in need of life-long treatment. We found that the Notch2/Delta-like Notch ligand 3 (DLL3) signaling pathway was active in GHoma tumorigenesis, progression, and invasion.

The γ-secretase inhibitor DAPT is of potential use in GHoma treatment targeting Notch signaling.

## Introduction

Growth hormone-producing adenomas (GHomas) can lead to acromegaly and the increased secretion of insulin-like growth factor 1 (IGF-1) (1). GHomas are currently treated with somatostatin receptor ligands and GH antagonists, which control growth hormone (GH) and IGF-1 levels (2). The pathogenesis of GHoma is largely unknown. Recurrent *GNAS* mutations have been identified in GHoma, but the clinical relevance of genetic changes has not yet been confirmed (3–5). The somatic landscape of GHoma can potentially influence Ca^2+^ and ATP pathways known to be involved in the GHoma tumorigenesis (6), gluconeogenesis, and glycolysis, mitochondrial dysfunction, oxidative stress, the cell cycle, and signaling pathways affecting MAPKs, TP53, VEGF, and inflammation (7–9).

The Notch signaling comprises a highly conserved pathway involved in determining cell activity, differentiation, and fate in both normal and tumor cells. It is initiated by the interaction of one of four Notch receptors with a number of possible ligands (10, 11). Different Notch receptors may have opposing functions within a single type of tumor. For example, Notch1 and Notch2 have antagonistic effects on the growth of embryonal brain tumor cell lines (12), and the anticancer activity of Notch inhibitors has been evaluated in clinical trials (13–15). Notch signaling is activated early in pituitary organogenesis and is required for the development of somatotrophs, lactotrophs, thyrotrophs, and corticotrophs (16). Notch3 expression is moderately elevated in non-functional pituitary adenomas (NFPAs) compared with that in functional adenomas including GHoma and prolactin-secreting adenomas (PRL) (17), but associated effects of Notch signaling have not been described.

Proteomic analysis complements the findings of RNA microarrays, and integrative analysis of the data is helpful for understanding the complex mechanisms influencing protein expression.

In this study, gene microarray analysis and nano-liquid chromatography–tandem mass spectrometry (nanoLC-MS/MS) of GHoma tumor tissue and GHoma cell lines were used to investigate the association of aberrant activation of Notch signaling with GHoma proliferation, invasiveness, and recurrence. Notch inhibitors suppressed tumor progression and GH release *in vitro* and *in vivo*.

## Materials and Methods

### Patients and Tissue Specimens

The medical records of patients treated for pituitary adenomas at the Department of Neurosurgery, Beijing Tiantan Hospital, Capital Medical University between May 2008 and July 2013 included 76 histologically confirmed GHomas. The 2007 World Health Organization classification was followed (18). The diagnostic criteria of invasive GHoma included: Knosp grade III–IV tumors and Hardy classification invasive adenomas; tumor cells pathologically confirmed as invading sellar bone or adjacent dura mater; and tumor cells invading the sphenoid sinus cavity or peripheral vascular and nerve.

Recurrence was diagnosed as the finding of a new histologically confirmed tumor in 20 patients. The study was conducted following approval of the protocol by the institutional review board. Written consent was obtained from all patients after being informed of the purpose of the research. Six normal pituitary samples were obtained by body donation. Tissue samples were frozen in liquid nitrogen for mRNA and protein isolation, fixed for pathological examination, or freshly harvested for primary cell culture.

This study was approved by the ethics committees of the Beijing Tiantan Hospital Affiliated to Capital Medical University (KY2013-015-02). Informed consent was obtained from all of the enrolled subjects, and the study was performed in full compliance with all principles of the Helsinki Declaration.

### Cell Culture

The GH3 rat pituitary cell line was purchased from China Institute of Cell Line Resources and cultured in phenol red-free Dulbecco's Modified Eagle's Medium (DMEM, Invitrogen, China) supplemented with 10% fetal bovine serum (Gibco, Auckland, USA) in a humidified incubator at 37°C with 5% CO_2_. Notch2-targeted shRNA (sc-40135) was obtained from Santa Cruz Biotech (Santa Cruz, CA, USA). Transfection was performed using lipofectamine 3000 (Invitrogen, Carlsbad, CA, USA) following the manufacturer's instructions. One microgram plasmid was used for transfecting 1 × 10^6^ GH3 cells and 3 × 10^5^ primary tumor cells.

For primary cell culture, samples were mechanically disrupted, filtered through a 70 μm cell strainer, washed with phosphate buffered saline (PBS), and cultured in complete DMEM medium supplemented with basic fibroblast growth factor (10 ng/ml), nerve growth factor (10 ng/ml), and L-glutamine (0.5 mM). γ-secretase inhibitor, DAPT (sc201315), was purchased from Santa Cruz Biotechnology (Dallas, TX, USA).

### Microarray Hybridization

Total RNA was isolated and purified using TRIzol reagent (Ambion, Thermo Fisher Scientific, USA) and assayed using an Agilent 2100 Bioanalyzer (Agilent Technologies, Santa Clara, CA, USA). Samples with a 28S to 18S rRNA ratio ≥ 0.7 number and 2100 RNA integrity (RIN) ≥ 7.0 were used to generate labeled targets. Total RNA was amplified and labeled with a One-Color Low Input Quick Amp Labeling Kit (Agilent Technologies, Santa Clara, CA, USA) following the manufacturer's instructions. Labeled complementary RNA (cRNA) was purified with an RNeasy Mini Kit (QIAGEN, Germantown, MD, USA). Each slide was hybridized with 1.65 μg Cy3-labeled cRNA using a Gene Expression Hybridization Kit (Agilent Technologies, Santa Clara, CA, USA) and a hybridization oven (Agilent Technologies, Santa Clara, CA, USA). After 17 h of hybridization, the slides were washed in staining dishes (Thermo Fisher Scientific, Waltham, MA, USA) using a Gene Expression Wash Buffer Kit (Agilent Technologies, Santa Clara, CA, USA) and scanned with a microarray reader (Agilent Technologies, Santa Clara, CA, USA) using default settings with a green dye channel, 5 μm scan resolution, and PMT settings of 100, 10, and 16% bits. The data were extracted using the Feature Extraction software package (version 10.7, Agilent), the raw data were normalized against the Quantile algorithm supplied in the GeneSpring software package (version 11.0, Agilent), and analyzed with the SBC Analysis System (version 2.9) software package (Shanghai Biotechnology Corporation, Shanghai, China). The significance threshold criteria were a false discovery rate (FDR) of < 0.05 and a fold-change > 2.

### Protein Preparation and nanoLC-MS/MS Analysis

Total protein from all five GHomas patients in Table S1 and five normal pituitary glands were extracted with a commercially available kit (Millipore, Billerica, MA, USA) as previously described and equal volumes were combined into a single pool (19). A 100 μg aliquot of each pooled sample was denatured, reduced, and alkylated with 4 μl reducing reagent at 60°C for 1 h and 2 μl cysteine blocking reagent at room temperature for 20 min following the iTRAQ protocol (Applied Biosystems) before overnight digestion with 2 μg trypsin at 37°C. The peptides were labeled with iTRAQ tags 118 or 117 (AbSciex). After labeling, the samples were combined, fractionated by chromatography, dried by vacuum centrifugation and combined in a single tube. A total of 48 fractions were collected, dried, and combined into 10 fractions following the strong cation exchange (SCX) chromatogram. Each fraction was injected into a desalting column (0.35 × 0.5 mm, 3 μm C18, 120 Å) and separated in an analytical column (75 μm × 150 mm, 3 μm C18, 120 Å) using an Eksigent nano-LC instrument (Eksigent, Dublin, CA, USA). The samples separated by capillary high-performance liquid chromatography were analyzed with a Triple TOF 5600+ system (AbSciex). Protein identification and proteome annotation were performed with ProteinPilot software (Applied Biosystems), and screened against the SwissProt database (March, 2013) using the Mascot 2.2 search engine (Matrix Science, London, UK).

### Ingenuity Pathway Analysis (IPA)

The differentially expressed genes/proteins were enriched with IPA software (Ingenuity Systems, Redwood City, CA, USA; www.ingenuity.com). And the canonical pathways were identified with the Core Analysis module included in the IPA library.

### Immunohistochemistry

Immunohistochemistry was conducted by Tissue microarray analysis (TMA) of 76 specimens as previously described (20) following evaluation of tumor content and quality in hematoxylin and eosin-stained sections. TMAs were processed in a Leica BOND-III (Leica Biosystems, Nussloch, Germany) automated, continuous random, access slide-staining system that simultaneously processes multiple immunohistochemistry (IHC) assays. Protocol F was selected, with 3 min of heat-induced epitope retrieval and Bond Polymer Refine Detection (Leica Biosystems, DS9800) of primary antibodies. Anti-Notch1, anti-Notch2, anti-Notch3, anti-Notch4, anti-DLL1, anti-DLL2, anti-DLL3, and anti-SSTR2/5 (Abcam, Cambridge, UK) were the primary antibodies. Expression was assayed in photographs taken with an Aperio AT2 whole slide scanning system (Leica Biosystems). Staining intensity was scored as 0, no staining; 1, weak; 2, moderate; and 3, strong staining. An H-score was calculated from the percentage of positively stained cells at each intensity level using the following formula: [1 × (% weakly stained cells) + 2 × (% moderately stained) + 3 × (% strongly stained cells)].

### Cell Viability and Migration Assays

GH3 cells were transfected with shRNA or empty control vectors (Non-effective 29-mer scrambled shRNA cassette in pGFP-C-shLenti Vector). GH3 cells or primary tumor cells were treated with a γ-secretase inhibitor for 24 h. Cultures were adjusted to a density of 1 × 10^5^ cells/ml, and 100 μl of cell suspension was plated into each well of a 96-well-plate and cultured for 0, 24, or 48 h before adding 20 μl MTS tetrazolium solution to each well with incubation for an additional 4 h. Absorbance at 490 nm was measured using an ELISA plate reader (Thermo, USA). Cell proliferation was also measured by Bromodeoxyuridine (BrdU) assays (KeyGen BioTech, Jiangsu, China). The percent of BrdU positive cell was based on cell count at five random fields (100×).

Cell migration was assayed on fibronectin- and Matrigel-coated polycarbonate filters in modified Transwell chambers (Corning, USA). GH3 cells or primary tumor cells (5 × 10^4^ cells) were introduced into the upper chambers. The time of incubation in the chambers was 24 h. Cells adhering to the lower membrane surface were fixed in 4% paraformaldehyde and stained with hematoxylin (Zhongshan Company, Beijing, China). The average number of migrated cells in five randomly chosen high-power fields was determined under light fields with fluorescent microscope (ZEISS, Jena, Germany). The assays were performed in triplicate.

### Quantitative Reverse-Transcription Polymerase Chain Reaction (qRT–PCR)

Total RNA was extracted from 24 frozen samples weighing ~10 mg using TRIzol reagent (Qiagen), and quantitative real-time (qRT)-PCR was performed as previously described (21), with an Applied Biosystems 7500 Fast System (Life Technologies, Carlsbad, USA). The fold-change in differential expression for each gene was calculated using the comparative CT method (2^−ΔΔCT^ method) as previously described (21). GAPDH was the housekeeping gene.

### Immunoblotting

Ten milligrams of 16 patient specimens and six xenograft samples were lysed in pH 7.4 TNE buffer (50 mM Tris–HCl, 150 mM NaCl, 1 mM EDTA; all from Sigma-Aldrich) containing 1% NP-40 (Calbiochem) with protease and phosphatase inhibitor cocktails (Roche). Total protein isolates were centrifuged at 12,000 g for 30 min at 4°C. Protein concentration was determined with a bicinchoninic acid (BCA) assay kit (Thermo Fisher Scientific). For immunoblotting assays, 40 μg of total protein was loaded onto 4–12% Bis-Trissodium dodecyl sulfate polyacrylamide gel electrophoresis (SDS-PAGE) gels, separated electrophoretically, and blotted onto polyvinylidene fluoride (PVDF) membranes. The blots were incubated with primary antibodies against DLL1, DLL3, DLL4, Notch1, Notch2, Notch4 (1:2,000, Abcam), VEGF (1:1,000, Abcam), and GAPDH (1:8,000, Sigma) followed by secondary antibodies tagged with horseradish peroxidase (1:5,000, Zhongshan Company). Blots were visualized by enhanced chemiluminescence, and densitometry was performed with an Amersham 600 Imager (GE).

### Magnetic Resonance Imaging (MRI)

MRI was performed with a 7.0T vertical bore Bruker nuclear magnetic resonance spectrometer (Bruker BioSpin, Rheinstetten, Germany). The scan parameters were optimized for gray-white matter contrast, with a T2-weighted 3D fast spin-echo sequence, TR = 2,000 ms, echo train length = 6, TEeff = 42 ms, field-of-view = 25 × 28 × 14 mm, and matrix size = 450 × 504 × 250, giving an image with 56 μm isotropic voxels. Total imaging time was 7 min.

### Transmission Electron Microscopy (TEM)

Specimens was fixed in 2.5% glutaraldehyde in pH 7.4 phosphate buffer for 6 h with shaking, washed in 0.1 M phosphate buffer, and then cut into 1 cm^3^ blocks. The blocks were post-fixed in 1% osmium tetroxide for 2 h at 4°C, washed three times in distilled water, dehydrated in a graded 50–100%ethanol series and propylene oxide, infiltrated with Epon 812, and polymerized for 48 h at 65°C. Thin sections were cut on an ultramicrotome using a diamond knife, collected on copper grids, and stained with 4% uranyl acetate and Reynold's lead citrate before observation with a JEM-1230 TEM.

### Mouse Xenograft Model

Animal experiments were performed using 6-week-old male athymic immune-deficient nude mice (SCXK2012-0001). Groups of five animals each were housed in an animal room at 23 ± 2°C, 55 ± 5% humidity, and a 12-h light, 12-h dark cycle. The mice were fed a standard, unrestricted diet. GH3 cells were harvested, re-suspended in PBS at 1 × 10^7^ cells/ml, and 200 μl of the cell suspension was injected into the flanks of mice on day 0. Intraperitoneal injection of 1 or 5 mg/kg body weight DAPT or PBS was administered daily. Tumors measured with calipers, and the volumes were calculated as (3.14 × length × width × depth)/6. After 15 days, the mice were euthanized, and the tumors were removed. All animal experiments were approved by the Animal Care and Use Committee of Beijing Neurosurgical Institute.

### Statistical Analysis

Chi square and Fisher's exact tests were used to determine the significance of differences in categorical variables, and the Chi square test was used to determine the significance of relationships between Notch/DLL expression and clinicopathological characteristics. One-way ANOVA was used to test the significance of differences in expression of Notch/DLL pathways in GH3 cells. *P* < 0.05 were considered to be significant.

## Results

### Clinical Characteristics of GHoma Patients

The 76 GHoma cases included 31 men and 45 women with an average age of 39.1 (range, 13–69) years. The mean tumor size was 4.74 ± 6.95 cm^3^ (range, 0.004–48.67). 35 were invasive GHomas, 41 were non-invasive GHomas according to their Knosp classification and intraoperative findings. The GH levels were 27 ± 14.17 ng/ml in the invasive and 15.69 ± 10.96 ng/ml in the non-invasive GHomas (*p* = 0.003, Table S2). Dense granules were seen in a greater proportion of non-invasive (26/41) than in invasive (14/35) GHomas (*p* = 0.042, Table S2), with ultra-structural characteristics including prominent, enlarged, tortuous, and multifocal Golgi bodies and an enlarged, abundant endoplasmic reticulum. The 5-year recurrence rates were 14 of 35 invasive tumors and 6 of 41 non-invasive tumors (*p* = 0.012).

### GHoma-Related Gene and Protein Expression

**Figure 1:**
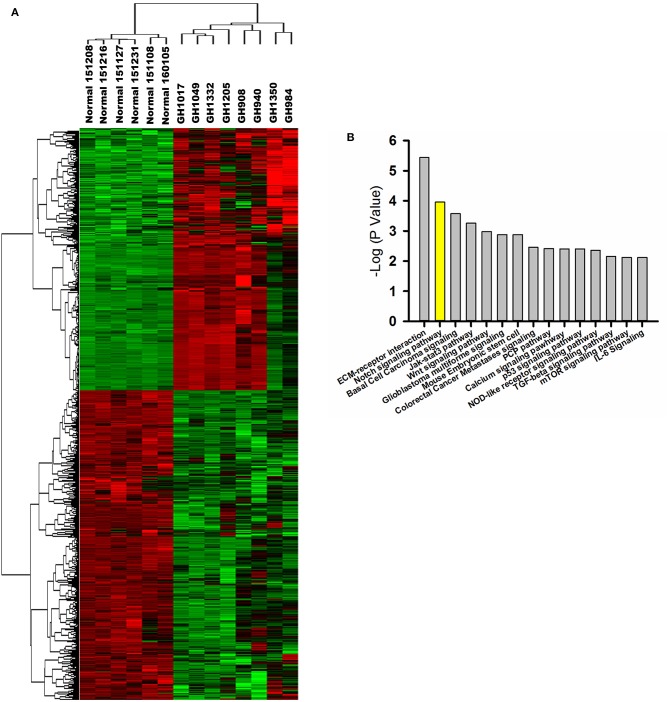
Gene expression profiling and proteomics analysis. **(A)** A heatmap of differentially expressed mRNAs (FC > 2 and FDR *q*< 0.1) between GHoma and normal pituitary tissue analyzed by hierarchical clustering. Each row represents a single mRNA. Each column represents an individual sample. High expression was shown in red; low expression was shown in green. **(B)** The top 15 canonical pathways from proteomics and transcriptome were shown.

Based on gene expression profiling, a heatmap created by two-dimensional hierarchical clustering revealed two major clusters, one containing six normal pituitary samples and the other containing eight GHoma samples (Figure 1A). A total of 4,179 genes was differentially expressed between the two clusters (*p* < 0.05 and FDR *q* < 0.01, and fold-change > 2.0 or < 0.5). Of these, 1,784 genes were upregulated and 2,395 genes were downregulated in GHoma. There was more than a ten-folds difference in the 347 genes expression of GHoma, upregulation in 203 and downregulation in 144.

Differentially expressed proteins were identified by nanoLC-MS/MS among five normal pituitary and five GHoma samples. Proteomics analysis identified 46,383 peptides that mapped to 5,083 proteins. A total of 349 proteins were differentially expressed (*p* < 0.05, with an iTRAQ ratio >2 or <0.5); 266 were upregulated and 83 were downregulated in GHomas.

The biological, cellular, and molecular functional characteristics of the differentially expressed genes and proteins were investigated by gene ontology analysis. The differentially expressed genes were identified to be related to biological processes such as H_2_O_2_ catabolism, syncytium formation, and cell differentiation. The differentially expressed proteins were associated with cell death and survival, protein synthesis, cellular growth and proliferation, and cell-to-cell signaling and interaction.

### IPA of Signaling Pathways

The core analyses of IPA were performed using the gene expression microarray and proteomics datasets. The top 15 canonical pathways were significant in the two datasets [*p* < 0.05, -log (*p*-value) > 1.3; Figure 1B]. The Notch signaling pathway was associated with GHoma and activated in GHoma, including 17 differentially expressed molecules (Table S3 and Figure S1).

### Notch Signaling Pathway Was Associated With GHoma Invasiveness

**Figure 2:**
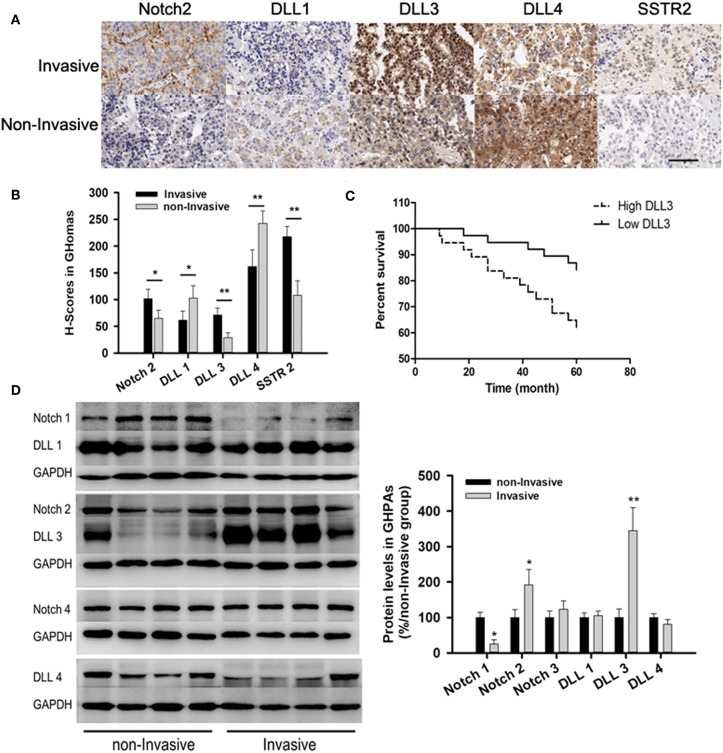
Notch signaling pathways in GHomas. **(A)** Expression of Notch2, DLL1, DLL3, DLL4, and SSTR2 in invasive and non-invasive GHomas. Brown: positively stained. **(B)** H-scores of Notch2, DLL1, DLL3, DLL4, and SSTR2. *P*-values was indicated by * and ** (<0.05 and <0.01). **(C)** Disease-free survival of patients was indicated to be significantly different according to the DLL3 expression. **(D)** Expression of Notch signaling between invasive and non-invasive GHomas. There was significant difference of Notch1, Notch2, and DLL3 between invasive and non-invasive based on the density value of bands. Assays were performed in triplicate.

**Figure 3:**
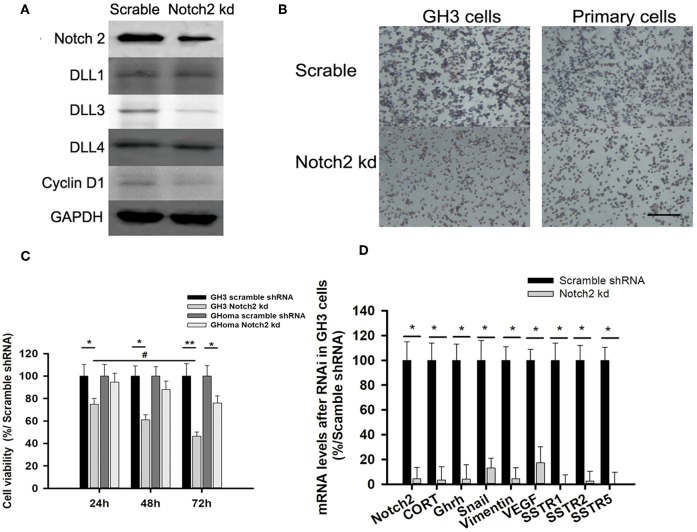
Notch2 knockdown inhibited proliferation and migration of GH3 and primary GHoma cells. **(A)** Expression of Notch pathways after Notch2 knockdown in GH3 cells. Notch2, Cyclin D1, and DLL3 expression was reduced to 31.3, 54.7, and 37.4% of those in the scramble group after Notch2 knockdown. **(B)** The Effect of Notch2 shRNA on cell migration. The average number of migrated GH3 cells in knockdown group was 27.3 ± 8.4% compared to that in the scramble group by transwells experiment, and the migrated primary tumor cells was 38.9 ± 13.1%compared to that in the scramble group. **(C)** The Effect of Notch2 shRNA on cell viability. The reduction of cell viability of GH3 was time dependence. **(D)** Expression of genes associated with migration, invasion, and growth hormone synthesis. The mRNA of CORT in knockdown group was 0.032-folds of that in scramble group, and GHRH 0.04-folds, Snail 0.13-folds, Vimentin 0.042-folds, VEGF 0.172-folds, SSTR1 0.005-folds, SSTR2 0.0245-folds, and SSTR5 0.006-folds. Assays were performed in triplicate. *P*-values indicated by * and ** (< 0.05, < 0.01), ^#^ (< 0.05).

Immunohistochemistry (IHC) from TMA showed the expressions of Notch2 (101.5 ± 18 vs. 65 ± 15) and its ligand DLL3 (71.3 ± 13 vs. 29 ± 9.2) were higher in invasive samples compared with those in non-invasive samples. IHC indicated the expressions of DLL1 (61.5 ± 17 vs. 102.8 ± 23) and DLL4 (162 ± 31 vs. 242.8 ± 23) were lower in invasive compared to those in non-invasive samples (Figures 3A,B). Additionally, somatostatin receptor type two (SSTR2) expression, which was long-acting somatostatin analogs widely used to treat acromegaly, was nearly two-fold higher in invasive (217.7 ± 19) than those in non-invasive samples (108 ± 27.2, *p* < 0.01; Figures 2A,B).

Patients with low DLL3 expression had significantly longer disease-free survival than those with high DLL3 expression (*P* = 0.027; Figure 2C).

Immunoblotting confirmed the expression of Notch2 and DLL3 were 1.92 ± 0.43 and 3.45 ± 0.65-folds higher in invasive than that in non-invasive samples (*p* < 0.05; Figure 2D).

### Notch2 Knockdown Inhibited Cell Migration and Invasion

As the activated Notch signaling was associated with invasive GHoma, the effect of Notch2 shRNA on the invasion was investigated in GH3 cell cultures. As shown in Figure S2, Notch2 expression in sh-B fragment was reduced to 31.3% of that in the negative control. Notch2 knockdown also reduced DLL3 and cyclin D1 expression, suggesting that cell cycle may be responsible for the decrease in cell proliferation (Figure 3A). Compared with the negative control, Notch2 knockdown significantly inhibited migration and cell viability of both GH3 cells and primary GHoma cells (Figures 3B,C). MTS experiment showed the cell viability was reduced to 74 ± 5.9, 61 ± 5.3, and 46.4 ± 4.6% in GH3 cells, and 94.5 ± 8.2, 88 ± 8.1, and 76 ± 7.4% in primary tumor cells after 24, 48, and 72 h knockdown of Notch2. And BrdU assays were used to confirm the effects of Notch2 shRNA or DAPT treatment on GH3 cells proliferation. The viability of GH3 cells was measured after 72 h Notch2 shRNA transfection or DAPT treatment. The percent of BrdU positive cells was 42.3 ± 6.7% in control group, and 38.1 ± 5.4% in vector scramble group, 17.6 ± 4.6% in sh-B group and 23.2 ± 4.6% in DAPT group, respectively, shown in Figure S3.

The mRNA expression of genes associated with cell migration and invasion and GH synthesis including Snail, vimentin, VEGF, GH releasing hormone (GHRH), cortactin, and SSTR1/2/5 were downregulated in GH3 cells after Notch2 knockdown (Figure 3D), but the expression of matrix metalloproteinase MMP2 and MMP9 mRNA was not affected by Notch2 knockdown (data not shown).

### Inhibition of γ-Secretase by DAPT Slowed Tumor Progression

**Figure 4:**
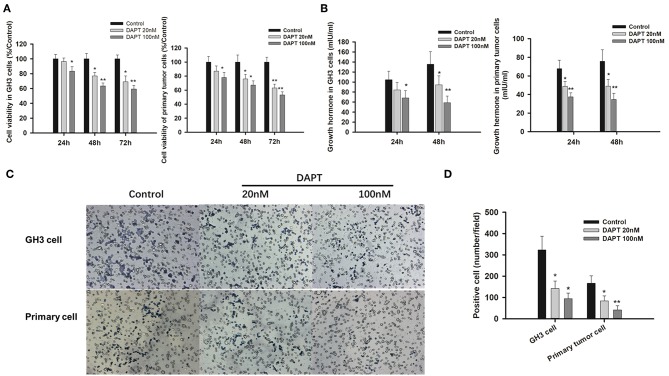
DAPT inhibited cell proliferation, growth hormone secretion, and invasion *in vitro*. **(A)** DAPT reduced the viability of GH3 cells and primary GHoma cells. The cell viability was reduced to 83.4 ± 7.6, 63.5 ± 5.3, and 59.4 ± 4.7% after 24, 48, and 72 h of 100 nM DAPT in GH3 cells, and 78 ± 7.1, 67 ± 6.3, 53 ± 4.7% in primary GHoma cell. **(B)** DAPT decreased growth hormone secretion in GH3 cell and primary GHoma cells. The levels of Growth hormone in GH3 cell culture were reduced to 84.3 ± 15 (20 nM) and 68.4 ± 14.5 (100 nM) mIU/ml after 24 h treatment, and 94.6 ± 18 (20 nM) and 58.9 ± 13 (100 nM) mIU/ml after 48 h treatment. The levels of Growth hormone in primary GHoma were 48.7 ± 5.3 (20 nM) and 48.9 ± 7.3 (100 nM) mIU/m after 24 h treatment, and 37.5 ± 4.2 (20 nM) and 34.7 ± 6.5 (100 nM) mIU/m after 48 h treatment. **(C)** DAPT inhibited the migration of GH3 cells and primary GHoma cells. **(D)** The average positive cells were reduced to 142 ± 35 and 94 ± 27 from 323 ± 64 after DAPT treatment in GH3 cell line, and 84 ± 23 and 42 ± 19 from 167 ± 35 in primary GHoma cells. Blue: positive cells. Assays were performed in triplicate. *P*-values indicated by * and ** (<0.05, <0.01).

As γ-secretase inhibitors have been reported to inhibit cell proliferation and enhance apoptosis in several cancer cell lines (21–23), the effects of DAPT on GHoma progression were investigated in GH3 cells and primary GHoma cells. The cell viability was reduced to 83.4 ± 7.6, 63.5 ± 5.3, and 59.4 ± 4.7% after 24, 48, and 72 h of 100 nM DAPT in GH3 cells and 78 ± 7.1, 67 ± 6.3, and 53 ± 4.7% in primary GHoma cells (Figure 4A). The levels of Growth hormone in GH3 cell culture were reduced to 58.9 ± 13 mIU/ml and 34.7 ± 6.5 mIU/m, respectively, after 48 h of 100 nM DAPT in GH3 cells and primary GHoma cells (Figure 4B).

The average number of migrated cells was reduced to 142 ± 35 and 94 ± 27 from 323 ± 64 after 20 and 100 nM DAPT in GH3 cell lines. There was the similar tendency in primary GHoma cells after DAPT treatment in Figures 4C,D.

**Figure 5:**
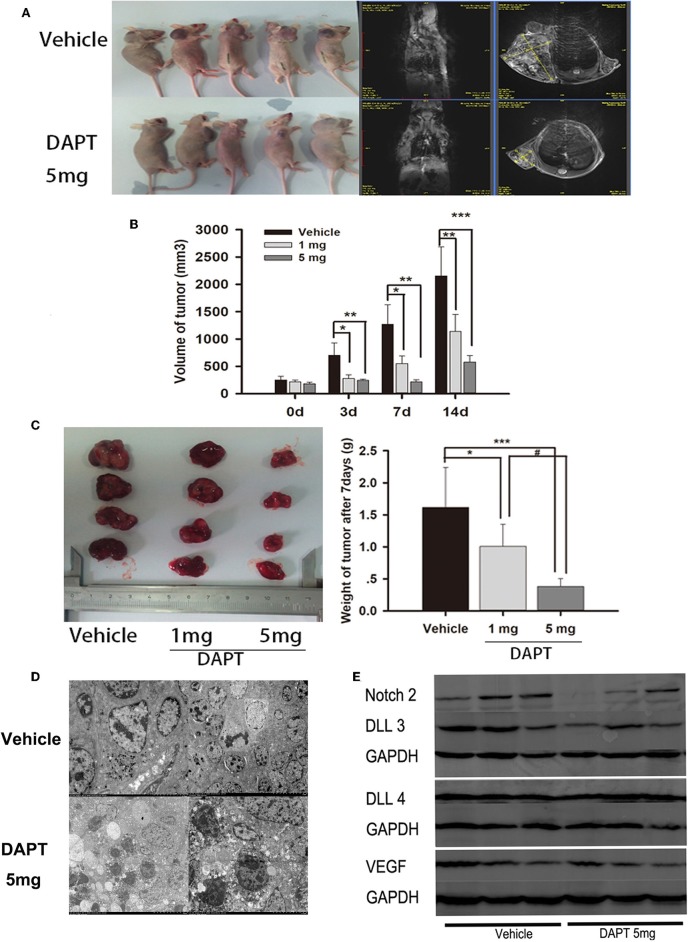
Effect of DAPT on xenografts growth *in vivo*. **(A)** Tumor-bearing mice and their representative MRI in upper control group and Tumor-bearing mice and their representative MRI in lower DAPT 5 mg-treated group. **(B)** Tumor volume change after DAPT treatment from day 0 to day 14. The tumor volume of DAPT 1 mg group was 39.5, 43.6, and 52.9% of control group volume, respectively. And the tumor volume of DAPT 5 mg group was 34.6, 16.8, and 23.5% of control group volume. **(C)** Tumor weight change after DAPT treatment from day 0 to day 14. The inhibition rate was 31.2% (1 mg) and 75% (5 mg) after DATP treatment. **(D)** Transmission electron microscopy of apoptotic cells after 5 mg DAPT treatment. TEM revealed less round particles in cytoplasm and more apoptotic cell after 5 mg DAPT treatment. **(E)** Expression of Notch signaling proteins after DAPT treatment. Compared to vehicle group, there were lower expression of Notch2, DLL3, and VEGF in tumors of 5 mg DAPT treated mice. *P*-values indicated by *, ** and *** (< 0.05, < 0.01, < 0.001), ^#^ (< 0.05).

In a mouse xenograft model established by subcutaneous injection of GH3 cells (Figure 5A), DAPT treatment significantly suppressed tumor growth (Figure 5B). The tumor volume of 1 mg group was reduced to 39.5, 43.6, and 52.9% compare to that of vehicle group after 3, 7, and 14 d. And the tumor volume of 5 mg group was reduced to 34.7, 16.8, and 26.8% compare to that of vehicle group after 3, 7, and 14 d (*p* < 0.01). The average tumor weight of the mice treated with 1 and 5 mg of DAPT were 68.8 and 25.0% of the weight in control mice, respectively (Figure 5C). TEM revealed abnormal mitosis, a large nuclear/cytoplasm ratio, nuclear anomalies, and the presence of large round particles in cytoplasm (Figure 5D). In order to identify the effect of DAPT on tumor progression by Notch2/DLL3 signaling pathway, Notch2/DLL pathways were detected by Western-blot. We found the Notch2 and DLL3 expressions were downregulated in DAPT-treated tumors compared to the vehicle groups (Figure 5E). And the DLL4 expression was not different between DAPT-treated tumors and the vehicle groups (Figure 5E). The results were consistent with DATP inhibition of GHoma proliferation and invasion after Notch2/DLL signaling blockade. Additionally, the VEGF expression was not different between DAPT-treated tumors and the vehicle groups (Figure 5E), which indicated the inhibition of DAPT on tumor progression was not dependent on VEGF signaling.

## Discussion

GHoma accounts for 10 to 20% of all pituitary tumors, causing acromegaly in adults and gigantism in adolescent (24), but the critical molecular events in GHoma progression have not been well-identified. As recurrent genetic mutations are rare, the analysis of dysregulated gene expression in GHoma is of particular value. Integrative analysis of transcriptomics and proteomics revealed several significantly altered pathways including Notch signaling in GHoma. Notch signaling was positively correlated with GHoma invasiveness. Notch pathways have distinct activities in various cancers. In the present GHoma study, Notch2/DLL3 signaling mediated enhanced invasiveness and frequent recurrence. The Notch ligand DLL3 has emerged as a novel therapeutic target in small cell lung cancer and high-grade neuroendocrine carcinomas (25). Epidermal growth factor-like domain multiple 7 (EGFL7), which modulates Notch2/DLL3 signaling, is involved in regulation of GHoma proliferation and invasiveness, and has been correlated with poor prognosis and tumor grade (26). Reduction of FSCN1 expression has been shown to downregulate Notch1 and DLL3 in GH3 cells (20). The finding in this study indicated that DAPT, an indirect Notch inhibitor, was able to suppress proliferation and invasion of GHoma *in vitro* and *in vivo* by blocking Notch2/DLLs signaling.

The Notch signaling pathway has been associated with epithelial-mesenchymal transition (EMT) in many types of cancer (27–29). EMT is closely associated with tumor cell migration and invasion, and overexpression of Snail and vimentin (30). That, together with the decreases of both Snail and vimentin following Notch2 knockdown, our data indicated that Notch2 may mediate EMT in GHoma development.

Notch signaling has multiple genes including at least four receptors and five ligands, three of which belong to the Delta family (10). The Notch2 and Notch3 receptors and many of their ligands and downstream genes are expressed during pituitary development. Emerging evidence also indicates an association of enhanced activation of Notch signaling with aggressive pituitary adenomas (31). The role of Notch2 in invasive GHomas was also proved in our study. Of the three DLL members, only DLL3 was significantly overexpressed in invasive GHoma and was associated with GHoma recurrence in human patients. This finding highlighted the role of Notch2/DLLs signaling in GHoma study.

The γ-Secretase cleaves Notch receptor and leads to activation of downstream genes. The γ-secretase inhibitors have shown antitumor activity both *in vitro* and *in vivo* (32, 33), and The γ-secretase inhibitors have been evaluated in clinical trials (13–15). DAPT has been shown to inhibit cell proliferation, migration, and invasion of gastric cancer by inhibiting the Notch1/Hes1 pathway, and in line with the study results, decreased expression of mesenchymal markers such as vimentin and Snail in gastric cancer (34). In this study, DAPT inhibited cancer growth both *in vitro* and *in vivo*, but Notch2/DLL rather than Notch1/Hes1 signaling was responsible for those effects in GH3 cells. The involvement of different pathways with similar effects of DAPT in different cancers is interesting. As DAPT does not directly influence Notch receptors, its effects may have resulted from expression of different Notch pathways. Lautaro et. al. recently found that DAPT could inactivate Notch signaling, especially Notch2, in GH3 inoculated nude mice, which was similar to our results (35).

Somatostatin receptor type two (SSTR2) is the predominant somatostatin receptor in GHomas, and somatostatin analogs specific for SSTR2, such as octreotide and lanreotide, are widely used to treat GH-producing tumors (36), but only about half the patients achieve even incomplete biochemical remission after being treated with these analogs, and the definition of resistance is controversial (37). The study results are consistent with increased SSTR2 levels in densely granulated GHoma (38), and SSTR2 expression was increased in cases of invasive GHoma. Notch1 promotion of somatostatin expression is accompanied by enhanced expression of the known SSTRs (39). In this study, Notch2 knockdown significantly decreased the expression of several SSTR moleculars, suggesting that crosstalk exists between the Notch and somatostatin signaling pathways.

In conclusion, Notch signaling was active in GHoma, particularly Notch2/DLL3 in invasive tumors. Notch2 mediated GHoma progression by modulating cell proliferation, migration and invasion. DAPT, a γ-secretase inhibitor, had GHoma antitumor effects, supporting its potential as a GHoma treatment, especially in patients with over-activation of Notch signaling and resistance to standard treatment.

## Author Contributions

HG conceived the idea. JF, JW, and YZ collected the samples, and performed proteomic analyses. LC, CLiu, LG, and DL established the cell model and performed *in vitro* experiments. QL, JL, QZ, XY, CLi, and YL performed the mouse xenograft model and *in vivo* experiments. HG, JF, and YZ interpreted the data. QZ, JF, and ZZ aided in the data analysis and wrote the manuscript. All authors approved the submission.

### Conflict of Interest Statement

The authors declare that the research was conducted in the absence of any commercial or financial relationships that could be construed as a potential conflict of interest.

## Abbreviations

cRNA: complementary RNA
DLL: Delta like canonical Notch ligand
DMEM: Dulbecco's Modified Eagle's Medium
EMT: epithelial-mesenchymal transition
FDR: false discovery rate
GH: growth hormone
GHoma: growth hormone-producing pituitary tumor
GO: Gene Ontology
IGF-1: insulin-like growth factor 1
IHC: immunohistochemistry
IPA: Ingenuity pathway analysis
LC-MS/MS: liquid chromatography tandem mass spectrometry
MRI: Magnetic resonance imaging
PBS: phosphate buffered saline
PVDF: polyvinylidene fluoride
qRT–PCR: Quantitative reverse-transcription polymerase chain reaction
TEM: Transmission electron microscopy
TMA: Tissue microarray.
