# Supplementary material for: DAPT, a γ-Secretase Inhibitor, Suppresses Tumorigenesis, and Progression of Growth Hormone-Producing Adenomas by Targeting Notch Signaling
Source: Front Oncol. 2019 Aug 27;9:809. doi: 10.3389/fonc.2019.00809 (PMC6718711; doi:10.3389/fonc.2019.00809)
Supplement: Supplementary file 1 [file Table_1.DOCX]

**Table S1 The Clinical features of 5 GHoma patients in LC-MS/MS experiment**

| **Case** | **Age (year)** | **Sex** | **Disease course (m)** | **Acromegaly** | **Headache** | **Vision** | **Maximum diameter (cm)** | **Growth hormone (ng/ml)** | **Resection degree** |
| --- | --- | --- | --- | --- | --- | --- | --- | --- | --- |
| **1** | 38 | F | 36 | yes | none | normal | 2.9 | 33.2 | subtotal |
| **2** | 28 | M | 60 | yes | none | normal | 2.3 | 18 | total |
| **3** | 57 | M | 120 | no | moderate | decrease | 3.1 | >40 | subtotal |
| **4** | 27 | F | 24 | yes | none | decrease | 3.7 | >40 | subtotal |
| **5** | 62 | F | 120 | no | moderate | decrease | 3.7 | 9.45 | total |
