# Supplementary material for: DAPT, a γ-Secretase Inhibitor, Suppresses Tumorigenesis, and Progression of Growth Hormone-Producing Adenomas by Targeting Notch Signaling
Source: Front Oncol. 2019 Aug 27;9:809. doi: 10.3389/fonc.2019.00809 (PMC6718711; doi:10.3389/fonc.2019.00809)
Supplement: Supplementary file 2 [file Table_2.DOCX]

**Table S2 The Clinical features of 76 GHoma patients**

| **Feature** | **GHomas** | |  |
| --- | --- | --- | --- |
|  | **invasive** | **non-invasive** |  |
| Sex |  |  | p=0.897 |
| Male | 14 | 17 |  |
| Female | 21 | 24 |  |
| Age |  |  | p=0.490 |
| ≥40 | 16 | 22 |  |
| <40 | 19 | 19 |  |
| Growth Hormone  (Media, ng/ml) |  |  | p=0.003 |
| ≥16.35 | 11 | 27 |  |
| <16.35 | 24 | 14 |  |
| Tumor size  (Median) |  |  | p=0.000 |
| ≥2.625 | 31 | 7 |  |
| <2.625 | 4 | 34 |  |
| TEM |  |  | P=0.042 |
| Dense granule | 14 | 26 |  |
| Loose granule | 21 | 15 |  |
