# Supplementary material for: DAPT, a γ-Secretase Inhibitor, Suppresses Tumorigenesis, and Progression of Growth Hormone-Producing Adenomas by Targeting Notch Signaling
Source: Front Oncol. 2019 Aug 27;9:809. doi: 10.3389/fonc.2019.00809 (PMC6718711; doi:10.3389/fonc.2019.00809)
Supplement: Supplementary file 3 [file Table_3.DOCX]

**Table S3 The details of Top fifteen pathway in GHomas from IPA analysis**
