# Supplementary material for: DAPT, a γ-Secretase Inhibitor, Suppresses Tumorigenesis, and Progression of Growth Hormone-Producing Adenomas by Targeting Notch Signaling
Source: Front Oncol. 2019 Aug 27;9:809. doi: 10.3389/fonc.2019.00809 (PMC6718711; doi:10.3389/fonc.2019.00809)

**Figure S2** Effects of different shRNA against Notch2 on protein expression in GH3 cells.

A: Western blots. B: Bands densities.

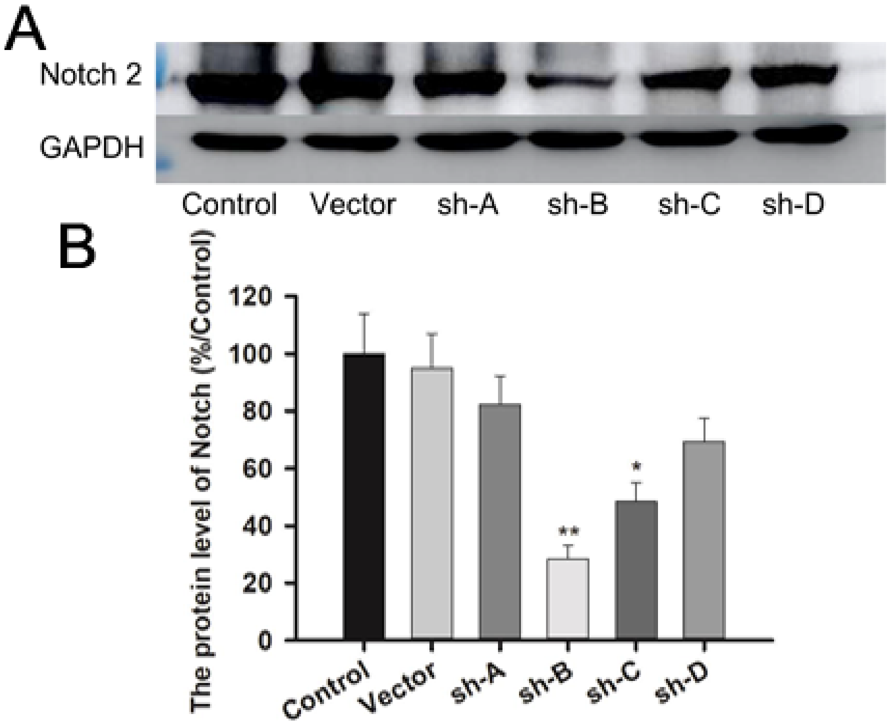

Supplement: Supplementary file 5 [file Image_2.pdf]
