# Supplementary material for: DAPT, a γ-Secretase Inhibitor, Suppresses Tumorigenesis, and Progression of Growth Hormone-Producing Adenomas by Targeting Notch Signaling
Source: Front Oncol. 2019 Aug 27;9:809. doi: 10.3389/fonc.2019.00809 (PMC6718711; doi:10.3389/fonc.2019.00809)

Figure S3 The Effect of shRNA or DAPT treatment on GH3 cells. A. Control, B: Vector C: shRNA with sh-B, D: DAPT (20nM).

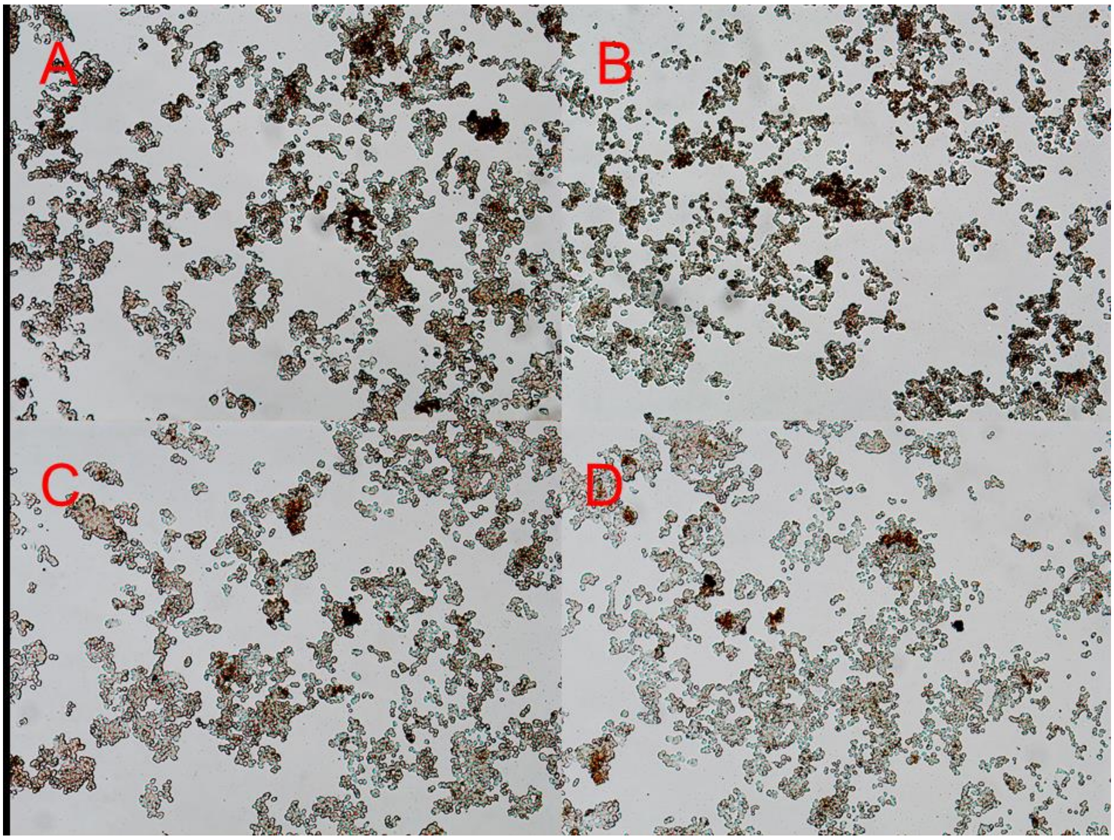

Supplement: Supplementary file 6 [file Image_3.pdf]
