# Supplementary material for: DAPT, a γ-Secretase Inhibitor, Suppresses Tumorigenesis, and Progression of Growth Hormone-Producing Adenomas by Targeting Notch Signaling
Source: Front Oncol. 2019 Aug 27;9:809. doi: 10.3389/fonc.2019.00809 (PMC6718711; doi:10.3389/fonc.2019.00809)

Figure S1 Notch pathways in GHoma according to IPA database. Upwards arrow: upregulation; Downwards arrow: downregulation.

.

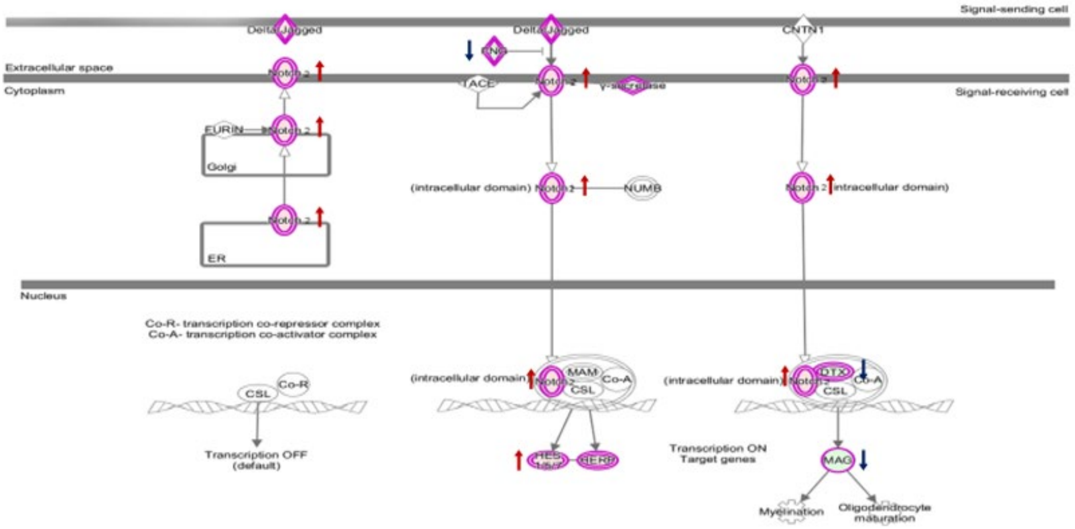

Supplement: Supplementary file 4 [file Image_1.pdf]
